# The change in Geriatric Nutritional Risk Index is associated with mortality in patients who start hemodialysis: Korean Renal Data Registry, 2016–2018

**DOI:** 10.1038/s41598-022-24981-1

**Published:** 2022-11-27

**Authors:** AJin Cho, Se Yeon Park, Yo Seop Cha, Hayne Cho Park, Do Hyoung Kim, Young-Ki Lee

**Affiliations:** 1 Department of Internal Medicine, Kangnam Sacred Heart Hospital, Hallym University College of Medicine, 1, Singil-Ro, Yeongdeungpo-Gu, Seoul, 07441 Korea; 2 Hallym University Kidney Research Institute, Seoul, Korea

**Keywords:** Diseases, Health care, Nephrology

## Abstract

Malnutrition is common in patients undergoing hemodialysis (HD) and is associated with mortality. This study aimed to investigate the association between changes in nutrition status measured by the Geriatric Nutritional Risk Index (GNRI) and all-cause mortality in patients who started HD. A nationwide retrospective cohort study was conducted based on the Korean Renal Data System database. Patients who started HD from January 2016 to December 2018, and were eligible for GNRI and GNRI trend were included. GNRI trend was a longitudinal change of GNRI, assessed by random slope in a mixed-effect model. Positive and negative random slopes in each patient were assigned to positive and negative GNRI trends. A total of 2313 patients were included and median follow-up period was 3.1 (2.6–3.7) years. GNRI values decreased over time (estimate − 1.212, 95% confidence interval (CI) − 1.116–0.692) and positive GNRI trend was associated with survival benefit (hazard ratio 0.55, 95% CI 0.36–0.84) after multivariate adjustment. These findings show that serial GNRI assessment, besides GNRI, is a useful prognostic factor for mortality in patients who start HD.

## Introduction

Malnutrition is a common condition in patients with end-stage renal disease (ESRD)^1,2^. In particular, protein–energy wasting (PEW) syndrome, which characterizes the loss of body protein mass and energy fuel, develops in this population and is associated with increased morbidity and mortality^3^. Identification of malnutrition and regular nutritional assessment are important in the management of hemodialysis (HD) patients.

There are several methods to assess the nutrition status of HD patients^4–7^. Among them, the Geriatric Nutritional Risk Index (GNRI), which uses body weight, height, and serum albumin, was developed as a simple method to assess nutritional condition^8^. Originally, it was developed to assess the nutrition status of elderly hospitalized patients. However, recent studies have shown that GNRI is a simple indicator of malnutrition where a lower GNRI is associated with cardiovascular events and mortality in HD patients^9–11^. Nutrition status of HD patients can change because they follow dietary recommendations for patients with ESRD, restricting products rich in potassium, sodium, and phosphates^12,13^. It is known that it is possible and reasonable to maintain an adequate protein intake by decreasing phosphate intake educating patients to choose protein sources with lower phosphate/protein ratios such as meats, and avoiding inorganic phosphate from ultra-processed foods.

In addition to baseline nutrition status, its trend might be an important prognostic factor for HD patients because their nutrition status can change over time after HD initiation. A recent study showed that annual changes in a nutritional marker predicted all-cause and cardiovascular mortality in patients undergoing maintenance HD. However, there are limited data on prognostic value of nutrition status change in HD patients. We hypothesized changes of nutritional marker can be a valuable prognostic factor for mortality in ESRD patients who start HD. In this study, we use GNRI as a marker for nutrition status and investigate impact of baseline GNRI and changes in GNRI on all-cause mortality.

## Results

### Patients’ characteristics by baseline GNRI

Table 1 shows baseline characteristics by median GNRI. Mean age of the study subjects was 62 ± 13 years, and 38.1% were women. There were 53.6% of patients who had diabetes as the primary cause of ESRD, and 70% used arteriovenous fistula as a vascular access for HD. Patients with GNRI ≥ 97.7 were younger and had a lower proportion of women, a higher proportion of arteriovenous fistula, higher values of hemoglobin, calcium, and phosphorus, and lower Kt/V.

**Table 1:** Baseline characteristics by median GNRI value.

	All (n = 2,313)	GNRI < 97.7 (n = 1155)	GNRI ≧ 97.7 (n = 1158)	p-value
Age at first HD, year	62 ± 13	66 ± 13	60 ± 13	< 0.001
Women	882 (38.1)	464 (40.2)	418 (36.1)	0.048
**Primary cause of ESRD**				0.064
Diabetes	1240 (53.6)	609 (52.7)	631 (54.5)	
Hypertension	495 (21.4)	247 (21.4)	248 (21.4)	
Glomerulonephritis	149 (6.4)	64 (5.5)	85 (7.3)	
Others	429 (18.5)	235 (20.3)	194 (16.8)	
SBP (mmHg)	142.7 ± 18.8	142.75 ± 19.2	142.4 ± 18.3	0.868
DBP (mmHg)	76.4 ± 11.8	76.2 ± 12.0	76.6 ± 11.5	0.394
Body mass index (kg/m^2^)	23.0 ± 3.6	22.0 ± 3.6	24.1 ± 3.3	< 0.001
**Comorbid conditions**				
Coronary artery disease	187 (8.1)	92 (8.0)	95 (8.2)	0.893
Heart failure	75 (3.2)	41 (3.5)	34 (2.9)	0.474
Arrhythmia	47 (2.0)	25 (2.2)	22 (1.9)	0.761
Cerebrovascular disease	142 (6.1)	74 (6.4)	68 (5.9)	0.653
Chronic pulmonary disease	5 (0.2)	0 (0.0)	5 (0.4)	0.074
Liver disease	84 (3.6)	47 (4.1)	37 (3.2)	0.311
GI disease	210 (9.1)	109 (9.4)	101 (8.7)	0.599
**Vascular access**				< 0.001
Fistular	1620 (70.0)	713 (61.7)	907 (78.3)	
Graft	341 (14.7)	175 (15.2)	166 (14.3)	
Catheter	352 (15.2)	267 (23.1)	85 (7.3)	
Kt/V	1.49 ± 0.29	1.51 ± 0.30	1.47 ± 0.27	< 0.001
Hemoglobin, g/dl	10.39 ± 1.10	10.20 ± 1.17	10.58 ± 0.99	< 0.001
Albumin, g/dl	3.83 ± 0.45	3.52 ± 0.37	4.15 ± 0.26	< 0.001
Calcium, mg/dl	8.54 ± 0.76	8.36 ± 0.74	8.72 ± 0.74	< 0.001
Phosphorus, mg/dl	4.68 ± 1.36	4.45 ± 1.29	4.91 ± 1.39	< 0.001
Creatinine, mg/dl	7.97 ± 2.74	7.37 ± 2.41	8.57 ± 2.92	< 0.001
GNRI	96.9 ± 6.91	90.27 ± 2.66	102.54 ± 3.67	< 0.001

Data are number (percentage) and mean ± standard deviation.

*HD* hemodialysis, *SBP* systolic blood pressure, *DBP* diastolic blood pressure, *ESRD* end-stage renal disease, *GI* gastrointestinal, *GNRI* geriatric nutrition risk index.

### Baseline GNRI and GNRI trend

**Figure 1:**
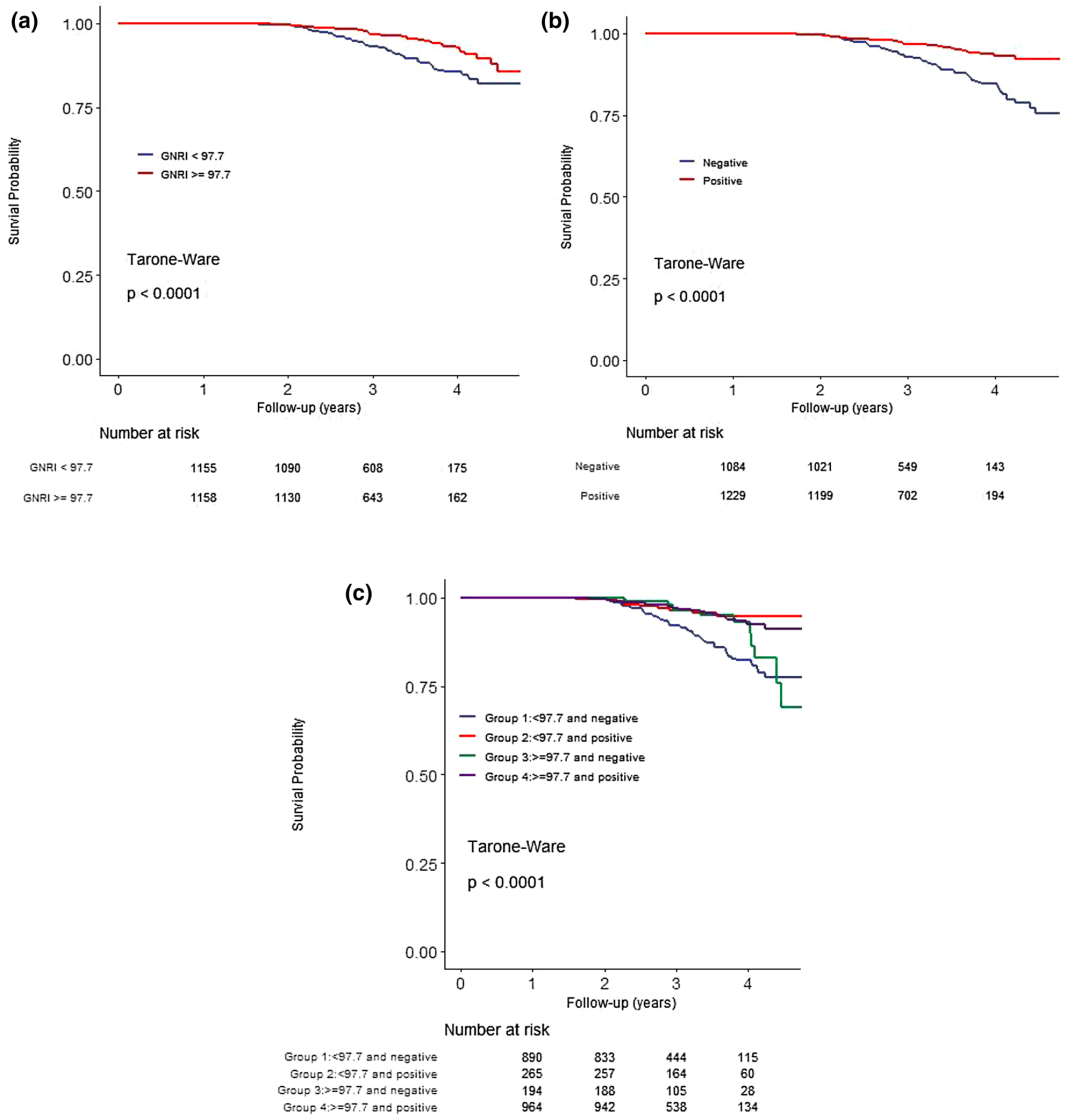
Kaplan–Meier survival curves for all-cause mortality. (**a**) GNRI < 97.7 vs. GNRI ≧ 97.7, (**b**) negative GNRI trend vs. positive GNRI trend, (**c**) Groups divided by baseline GNRI and GNRI trend. Group 1, GNRI < 97.7 and negative GNRI trend; Group 2, GNRI < 97.7 and positive GNRI trend; Group 3, GNRI ≧ 97.7 and negative GNRI trend; Group 4, GNRI ≧ 97.7 and positive GNRI trend. *GNRI* Geriatric Nutritional Risk Index.

Figure 1 shows the correlation between baseline GNRI and individual GNRI slope. Baseline GNRI was positively correlated with GNRI slope (r = 0.61, p-value < 0.001). Overall, GNRI values decreased over time (estimate − 1.212, 95% CI − 1.116 to 0.692). Changes of GNRI per year in each group were as follows: Group 1, − 1.395 (− 1.677 to − 1.113); Group 2, − 0.162 (− 0.272 to 0.052); Group 3, 1.318 (1.157 to 1.479); Group 4, 1.989 (1.738 to 2.24). Patients’ characteristics according to GNRI trend (positive and negative) are presented in Table 2. Among 1,158 patients with GNRI values ≥ 97.7, 194 patients (16.8%) had negative trends in GNRI. This group was older and had a higher proportion of women, lower values of serum phosphorus, and higher values of hemoglobin and Kt/V compared with the group with GNRI ≥ 97.7 and positive GNRI trend. In patients with GNRI < 97.7, 890 patients (77.1%) were included in the negative trend group that was older and had a higher proportion of women and lower values of phosphorus. Primary causes of ESRD and comorbid conditions were not different between the positive and negative groups.

**Table 2:** Baseline characteristics of the incident HD patients by GNRI trend.

	GNRI < 97.7 (n = 1155)			GNRI ≧ 97.7 (n = 1158)		
	Group 1 N = 890	Group 2 N = 265	Group 3 N = 194		Group 4 N = 964	p-value
Age at first HD, year	66 ± 13	59 ± 13	65 ± 11		59 ± 13	< 0.001
Women	384 (43.1)	80 (30.2)	96 (49.5)		322 (33.4)	< 0.001
**Primary cause of ESRD**						0.022
Diabetes	453 (50.9)	156 (58.9)	100 (51.5)		531 (55.1)	
Hypertension	201 (22.6)	46 (17.4)	34 (17.5)		214 (22.2)	
Glomerulonephritis	51 (5.7)	13 (4.9)	17 (8.8)		68 (7.1)	
Others	185 (20.8)	50 (18.9)	43 (22.2)		151 (15.7)	
SBP (mmHg)	142.0 ± 19.4	144.7 ± 18.4	1440.5 ± 18.7		142.8 ± 18.2	0.063
DBP (mmHg)	75.9 ± 12.2	77.3 ± 11.3	74.2 ± 11.0		77.1 ± 11.6	0.004
**Comorbid conditions**						
Coronary artery disease	74 (8.3)	18 (6.8)	15 (7.7)		80 (8.3)	0.861
Heart failure	35 (3.9)	6 (2.3)	5 (2.6)		29 (3.0)	0.457
Arrhythmia	21 (2.4)	4 (1.5)	5 (2.6)		17 (1.8)	0.686
Cerebrovascular disease	57 (6.4)	17 (6.4)	16 (8.2)		52 (5.4)	0.463
Chronic pulmonary disease	0 (0)	0 (0)	0 (0)		5 (0.5)	0.072
Liver disease	32 (3.6)	15 (5.7)	3 (1.5)		34 (3.5)	0.135
GI disease	84 (9.4)	25 (9.4)	17 (8.8)		84 (8.7)	0.949
**Vascular access**						< 0.001
Fistular	542 (60.9)	171 (64.5)	149 (76.8)		758 (78.6)	
Graft	145 (16.3)	30 (11.3)	31 (16.0)		135 (14.0)	
Catheter	203 (22.8)	64 (24.2)	14 (7.2)		71 (7.4)	
Kt/V	1.53 ± 0.31	1.42 ± 0.26	1.53 ± 0.28		1.46 ± 0.27	< 0.001
Hemoglobin, g/dl	10.20 ± 1.14	10.23 ± 1.28	10.74 ± 1.01		10.55 ± 0.98	< 0.001
Albumin, g/dl	3.49 ± 0.39	3.64 ± 0.26	4.00 ± 0.19		4.18 ± 0.26	< 0.001
Calcium, mg/dl	8.36 ± 0.75	8.36 ± 0.68	8.68 ± 0.80		8.72 ± 0.73	< 0.001
Phosphorus, mg/dl	4.41 ± 1.27	4.58 ± 1.34	4.58 ± 1.37		4.98 ± 1.38	< 0.001
Creatinine, mg/dl	7.19 ± 2.36	8.00 ± 2.49	7.67 ± 2.54		8.75 ± 2.96	< 0.001
GNRI	90.34 ± 5.92	94.39 ± 3.10	99.65 ± 1.83		103.12 ± 3.68	< 0.001

Data are number (percentage) and mean ± standard deviation.

*HD* hemodialysis, *SBP* systolic blood pressure, *DBP* diastolic blood pressure, *ESRD* end-stage renal disease, *GI* gastrointestinal, *GNRI* geriatric nutrition risk index.

### Association between GNRI and mortality

**Figure 2:**
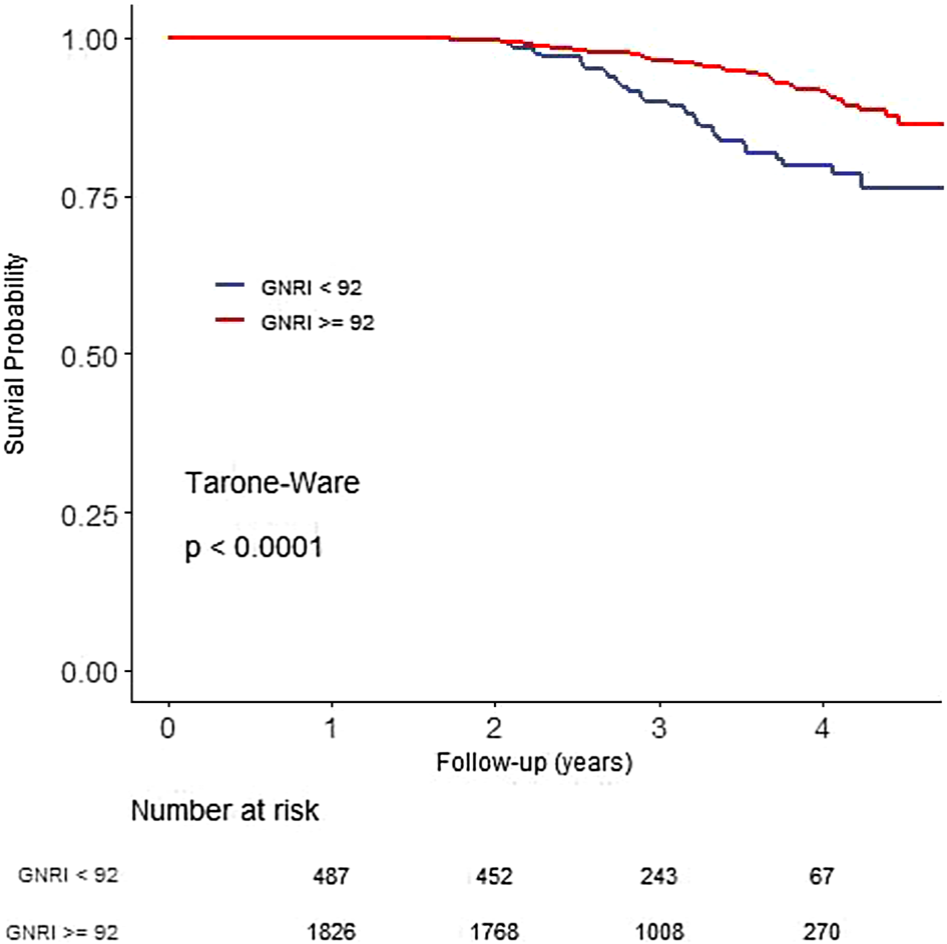
Kaplan–Meier survival curves for all-cause mortality: GNRI < 92 vs. GNRI ≧ 92. *GNRI* Geriatric Nutritional Risk Index.

The median follow-up period was 3.1 (2.6–3.7) years. There were 148 deaths during the study period, and 253 patients were lost to follow-up. Crude mortality rate was 2.04 per 100 person-years. Comparisons of survival rate between the groups are shown in Fig. 2. The 4-year survival rate showed significant differences between patients with baseline GNRI < 97.7 and ≥ 97.7 (82.6% versus 90.4%, *P* < 0.001), and patients with negative and positive GNRI trends (81.2% versus 91%, *P* < 0.001). The 4-year survival rates according to the groups were 78.4%, 91.3%, 87.9%, and 90.0% in Group 1, Group 2, Group 3, and Group 4, respectively (*P* < 0.001). In adjusted Cox proportional regression models, patients with positive GNRI trend showed a 45% mortality risk reduction compared with those with negative GNRI trend (HR 0.55, 95% CI 0.36–0.84, *P* = 0.005) (Table 3). Compared with Group 1, Group 2 (HR 0.38, 95% CI 0.2–0.74, *P* = 0.004) and Group 4 (HR 0.49, 95% CI 0.32–0.75, *P* < 0.001) were associated with a survival benefit in these models. We performed Cox proportional regression models according to baseline median GNRI 97.7 (Table 3). In patients with GNRI < 97.7, baseline GNRI (HR 0.96, 95% CI 0.92–0.99, *P* = 0.014) and GNRI trend (HR 0.43, 95% CI 0.22–0.88, *P* = 0.020) were associated with mortality. However, baseline GNRI and GNRI trend did not show a significant association in patients with GNRI ≥ 97.7. There is a significant association between GNRI and risk of mortality, with a unit decrease in the GNRI corresponding to 1.08-fold increase in risk of death (HR: 1.08, 95% CI 1.031–1.128). We further performed multivariable Cox regression analysis according to GNRI 92 which was suggested in other study^15^ as a cut-off value. GNRI ≥ 92 (HR 0.648, 95% CI 0.434–0.968, *P* = 0.034) and positive trend of GNRI (HR 0.596, 95% CI 0.399–0.889, *P* = 0.011) was associated with survival benefit. Survival curves between patients with GNRI < 92 and GNRI ≥ 92 demonstrated in Fig. 2.

**Table 3:** Cox-proportional hazard ratios (95% confidence intervals) for all-cause mortality.

	Unadjusted	p-value	Adjusted*	p-value
**All**				
GNRI ≧ 97.7 vs. 97.7	0.50 (0.35–0.69)	< 0.001	0.85 (0.55–1.30)	0.442
GNRI trend, positive vs. negative	0.39 (0.28–0.55)	< 0.001	0.55 (0.36–0.84)	0.005
**Groups (vs. Group 1)**				0.001
Group 2	0.32 (0.17–0.62)	< 0.001	0.38 (0.2–0.74)	0.004
Group 3	0.59 (0.33–1.05)	0.07	0.62 (0.34–1.14)	0.126
Group 4	0.37 (0.25–0.54)	< 0.001	0.49 (0.32–0.75)	< 0.001
**GNRI < 97.7**				
GNRI	0.94 (0.92–0.97)	< 0.001	0.96 (0.92 – 0.99)	0.014
GNRI trend, positive vs. negative	0.32 (0.17–0.62)	< 0.001	0.43 (0.22– 0.88)	0.020
**GNRI ≧ 97.7**				
GNRI	0.93 (0.86–1.01)	0.095	0.96 (0.87–1.05)	0.349
GNRI trend, positive vs. negative	0.65 (0.35–1.23)	0.19	0.84 (0.41–1.72)	0.634

*GNRI* geriatric nutrition risk index.

*Adjusted for age, sex, primary causes of ESRD, the presence of coronary artery disease, heart failure, arrhythmia, cerebrovascular disease, chronic pulmonary disease, liver disease, gastrointestinal disease, systolic blood pressure, diastolic blood pressure, Kt/V and laboratory findings (hemoglobin, calcium phosphorus and creatinine).

### Prediction of mortality and GNRI

Next, we performed C-index to assess whether the predictive accuracy of mortality would improve after the addition of GNRI trend to the baseline model, including the GNRI and risk factors (age, sex, primary causes of ESRD, the presence of coronary artery disease, heart failure, arrhythmia, cerebrovascular disease, chronic pulmonary disease, liver disease, gastrointestinal disease, and laboratory findings). AUCs for all-cause mortality were 0.737 (0.696–0.777) in the baseline model and 0.740 (0.701–0.78) in the addition model. The difference was not significant (*P* = 0.445). However, the NRI and IDI for all-cause mortality were significantly improved by the addition of GNRI trend to the baseline model (NRI, 0.36, *P* < 0.001; and IDI, 0.003, *P* = 0.018).

## Discussion

In this study, GNRI trend was a significant prognostic factor for all-cause mortality in incident HD patients. When we divided the patients according to median baseline GNRI, GNRI trends were not significantly associated with mortality in the group with GNRI ≥ 97.7. However, patients with baseline GNRI < 97.7 showed increased mortality as the GNRI changes showed negative trends throughout the study period. Compared with the group with GNRI < 97.7 and decreased trend of GNRI (Group 1), groups with an increased trend of GNRI (Groups 2 and 4) had higher survival rates despite baseline GNRI. This result suggests a change in nutrition status may be an important prognostic factor for mortality in incident HD patients with preexisting malnutrition. Malnutrition is a common condition and strongly associated with high mortality and morbidity in patients with ESRD^1,16–18^. Assessment and monitoring of nutrition status are essential to diagnose, treat, and prevent malnutrition in patients undergoing HD. GNRI is a simple and objective nutritional screening tool^8^. Previous studies have shown that GNRI is a significant predictor of mortality and cardiovascular events in patients undergoing HD and an effective screening tool for identifying malnutrition^9,10,19^.

Patients with ESRD with preexisting malnutrition at initiation of HD became vulnerable to deterioration of nutrition status on account of the dialysis procedure, suboptimal dietary intake, and uremia. About 3–7 g of albumin and 5–8 g of amino acid are lost during HD and it may contribute to chronic nutrient losses and hypoalbuminemia^20–22^. Globally 30–80% of HD patients are affected by inadequate dietary protein intake^23–25^. Dietary inadequacy in HD patients may be attributed to poor appetite, taste alteration, and monotonous dietary patterns^26^. HD treatment causes dialysis-associated inflammation. Several factors are relevant, such as direct effects of the dialysis membrane with stimulation of complement and cytokines, impure dialysate containing cytokine-inducing substances, and infected or old clotted dialysis access^27–30^. Some of these contributing factors are modifiable. Therefore, assessment of changes in nutrition status is a critical step to identify the factors of malnutrition etiology and to treat and prevent malnutrition accordingly.

There have been few studies investigating the effect of nutritional changes on mortality in HD patients. Pifer et al. prospectively investigated predictive values of changes in nutritional indicators for mortality risk in HD patients^31^. During a six-month follow-up, HD patients experiencing declines in body mass index and serum albumin levels had increased mortality risk. Yajima et al. reported that a lower baseline GNRI and declining GNRI in the first year were independently associated with increased risks of all-cause and cardiovascular mortality in patients undergoing maintenance HD^14^. Patients with lower baseline GNRI and declining GNRI had the highest risk of mortality. In a study including 75 HD patients, the changes in GNRI were associated with changes in nutritional scores, body composition parameters, and inflammation (interleukin-6 level) in HD patients^32^. The authors suggested both MIS and GNRI are valid tools for longitudinal observation of nutrition status of prevalent HD patients.

In the present study, patients with lower baseline GNRI and negative trend of GNRI had the lowest survival rate. In the group with lower baseline GNRI, a negative GNRI change was a significant predictor of mortality. However, the change did not have a significant association with mortality in the group with higher baseline GNRI. This suggests longitudinal assessment of HD patient’s nutrition status might be important for reducing mortality rate in patients with preexisting malnutrition. The results demonstrate that addition of GNRI change to a model with an established risk factor for mortality might improve predictability of survival. However, a predicting model with GNRI overestimates predictability improvement, so NRI and IDI tend to be seen significant. Further study is needed to assess predictive value of GNRI change for mortality in HD patients.

There are several limitations to this study. First, as a retrospective study, it is possible that model adjustment failed to adequately account for selected confounders, leaving residual confounding. And registry data lack detailed information. Furthermore, we included only the Korean HD population, which might not be generalizable to other populations. Therefore, the predictive validity of the GNRI should be examined in patients with ESRD from other countries of origin. Second, the follow-up time was not long enough and might have had an impact on the present results. The long-term association between GNRI trend and mortality in patients with ESRD needs further investigation, especially in terms of cardiovascular events. Third, 253 patients were censored during follow-up. When we compared demographic and clinical characteristics between the patients who were lost to follow-up (n = 253) or not (n = 1912), which were not significantly different except for age and primary cause of ESRD. The GNRI values (97.2 ± 7.3 vs. 96.6 ± 7.6, p-value = 0.20) and proportion of positive slope (55.2% vs. 49.4%, p-value = 0.10) were not significantly different between the two groups. Fourth, GNRI formula include albumin, which is influenced by non-nutritional factors, such as inflammation. Further, we could not compare GNRI with other nutritional screening tools such as MIS or Subjective Global Assessment. Finally, data in KORDS are updated yearly, therefore patients had only one GNRI at each year.

In conclusion, our findings show that low baseline GNRI and negative GNRI trend are associated with high all-cause mortality in incident HD patients. This suggests longitudinal nutrition assessments are important and GNRI might be a useful prognostic tool to predict survival.

## Methods

### Data source and study population

The Korean Society of Nephrology has maintained a nationwide ESRD registry of data from the Korean Renal Data System (KORDS), as the representative registry of the ESRD population in Korea since 1985. Detailed information on the KORDS can be found elsewhere^33,34^. This registry collects data based on voluntary enrollment via an online registry program ((http://www.ksn.or.kr) and is updated yearly at each dialysis center. We conducted a retrospective cohort study based on the KORDS database. This study included incident patients who started HD from January 2016 to December 2018 and had at least 3 years of follow-up period. 2313 patients who were available to provide a GNRI trend were included for outcome assessment and follow-up until the end of 2020 or study departure. The study was performed according to the Ethics of Clinical Research (Declaration of Helsinki) and approved by the Institutional Review Board of Hallym University Kangnam Sacred Heart Hospital (IRB No: 2021-04-036) and the KORDS Committee. We could not obtain informed consent from the patients because we used deidentified and retrospective data. This issue was also confirmed by the hospital’s Institutional Review Board.

### Measurements

Demographic data (age and sex), clinical information (primary cause of ESRD and comorbid conditions), and laboratory data (serum albumin, hemoglobin, phosphorus, and calcium) at initiation were collected. Blood samples were obtained prior to HD sessions. Comorbid conditions included coronary artery disease, heart failure, arrhythmia, cerebrovascular disease, chronic pulmonary disease, liver disease, and gastrointestinal disease. Cerebrovascular diseases included stroke, hypertension, and peripheral vascular disease. Liver diseases included hepatitis B and C, liver cirrhosis, and hemochromatosis. Gastrointestinal diseases included gastric and duodenal ulcers and constipation. Single-pool Kt/V, which represents HD efficacy, was determined using two-point urea modeling. The GNRI was calculated based on the following equation:^35^

$${\text{GNRI}}\, = \,\left[ {1.489\, \times \,{\text{albumin}}\,\left( {g/L} \right)} \right]\, + \,\left[ {41.7\, \times \,\left( {{\text{dry}}\,{\text{weight}}/{\text{ideal}}\,{\text{weight}}} \right)} \right].$$

We set dry weight/ideal weight = 1 when the actual dry weight was greater than the ideal weight. The ideal weight was calculated from the Lorenz equation, as follows^8^. For males, ${\text{Height}}\, - \,100\, - \,\left[ {\left( {{\text{height}}\, - \,150} \right)/4} \right]$. For females, ${\text{Height}}\, - \,100\, - \,\left[ {\left( {{\text{height}}\, - \,150} \right)/2.5} \right]$.

### Outcomes and GNRI trend

The outcome of interest in the present study was all-cause mortality. Patients lost to follow-up, transferred to another treatment modality or followed until the end of the study were censored. Random slopes in each subject were assessed to obtain GNRI trend in a mixed-effect model. Random slopes of zero or positive values were considered as a positive GNRI trend. Negative random slopes meant a negative GNRI trend. Patients were divided into four groups according to baseline median GNRI (higher baseline GNRI (≥ 97.7) and lower baseline GNRI (< 97.7)) and GNRI trends. Group 1 and Group 2 indicated patients with GNRI < 97.7 and negative and positive GNRI trend, respectively. Patients with GNRI ≥ 97.7 and negative were assigned to Group 3 and ones with GNRI ≥ 97.7 and positive were assigned to Group 4.

### Statistical analysis

Continuous variables were expressed as mean ± standard deviation, and categorical variables as the number and proportion of patients. The differences among the groups were compared using *t* test and the chi-squared test. GNRI trends were obtained by random slopes from a mixed-effect model in which GNRI and time were a response and an explanatory variable, respectively, and random effects (intercept and slope) were considered within individual patients. Kendall’s Tau was performed to analyze the correlation between GNRIs and GNRI slopes. The survival rate was estimated using the Kaplan–Meier method, and the difference was analyzed using the Tharone-Ware test. Cox proportional hazard models were used to estimate adjusted and unadjusted hazard ratios (HRs) and 95% confidence intervals (CIs) for all-cause mortality. The multiple regression model included all covariates such as baseline GNRIs and GNRI trends. In all these analyses, the proportional hazards assumption was tested by Schoenfeld residual method. We performed joint modelling with the longitudinal and time-to-event models. The JM package was used to fit the joint model^36^. Receiver-operating characteristic (ROC) curves were analyzed to determine the predictive accuracy of mortality between the baseline model with GNRI and the model with addition of GNRI trend as measured by the area under the curve (AUC). DeLong test was conducted to test differences between the ROC curves. The net reclassification improvement (NRI) was defined as a relative indicator of the number of patients with improved predicted mortality, and the integrated discrimination improvement (IDI) was defined as an average improvement in predicted mortality after the addition of GNRI trends to the baseline model. All *P* values were two-sided, and *P* < 0.05 was considered statistically significant. Statistical analyses were performed using R version 4.0.5 (R Foundation for Statistical Computing, Vienna, Austria. URL https://www.R-project.org/).

## Data Availability

The data presented in this study are available on request from the corresponding author.
